# Establishment of Classification of Tibial Plateau Fracture Associated with Proximal Fibular Fracture

**DOI:** 10.1111/os.12424

**Published:** 2019-02-08

**Authors:** Zhan‐le Zheng, Yi‐yang Yu, Heng‐rui Chang, Huan Liu, Hui‐lin Zhou, Ying‐ze Zhang

**Affiliations:** ^1^ Department of Orthopaedic Trauma Center, Key Biomechanics Lab of Hebei Province The Third Hospital of Hebei Medical University Shijiazhuang China

**Keywords:** Classification, Fibular, Fracture, Tibial plateau

## Abstract

**Objective:**

The purpose of this retrospective study was to determine the incidence of fibular fractures as an associated injury in tibial plateau fractures according to CT scan. We also attempt to introduce a new morphological sub‐classification on this associated injury and to analyze the correlation between this classification and tibial plateau fractures.

**Methods:**

We selected cases with fibular fractures from all the tibial plateau fracture patients. The cases were further divided into 2 groups: unicondylar group and bicondylar group. On the basis of our new classification system of fibular fracture, all the included cases were divided into 5 subgroups.

**Results:**

Finally, a total of 150 cases associated with fibular fractures in 502 tibial plateau fracture cases were identified from our institution database. The incidence of fibular head fracture in tibial plateau fractures was 29.88% (150/502). Seventy‐one cases (47.3%) were involved one condyle, and 79 cases (52.7%) involved both. It shows significant difference in the subgroup of avulsion fracture with horizontal fracture line (Type A) which is ratio of 16.9% in unicondylar group and 1.27% in bicondylar group.

**Conclusion:**

A new classification of this associated injury describing the morphology of the fracture fragments may improve operative planning.

Tibial plateau fractures are common injuries which constitute 1.6% of all fractures from both high‐energy and low‐energy trauma^1^. Hence, there are several fracture patterns. A number of classification systems have been proposed to categorize the fracture patterns, simplify communication in clinical practice, and provide guidelines for preoperative planning. The current widely recognized systems are the Schatzker classification and the OTA/AO classification^2^.

Tibial plateau fractures, especially comminuted fractures (Schatzker V and VI), are always associated with serious injuries. The injuries, such as compartment syndrome, neurovascular injury, and ligamentous disruption, have been widely reported^3^, ^4^, ^5^, ^6^, ^7^. Nevertheless, fibular fractures tend to be neglected in the literature as an associated injury of tibial plateau fractures. Zhu *et al.* reports an incidence of fibular head fractures in bicondylar tibial plateau fractures of 63.41%^8^. The proximal fibular zone is comprised of ligaments, tendons, the common peroneal nerve, and tibiofibular bony structure. Anatomically, the lateral collateral ligament and tendon of the long head of the biceps femoris muscle are attached to the lateral margin of the fibular head. In addition, the popliteofibular, arcuate, and fablelofibular ligaments attached to the fibular styloid process constitute the acute complex, which contributes to the posterolateral stability of the knee^9^. Proximal fibular dislocated fractures cause knee posterolateral complex (PLC) injuries, often creating obvious postural instability and external rotation instability^10^, ^11^, ^12^. There are many tibial fracture classifications, but none of them includes a classification for proximal fibular fractures. In our clinical practice, the importance of fibular fractures has always been realized, which has helped us to treat tibial fractures easily. Therefore, we wanted to conduct a study on tibial plateau fractures associated with proximal fibular fractures. The purpose of this study is: (i) to investigate the incidence and morphology of fibular fractures as an associated injury in tibial plateau fractures; (ii) to further clarify the importance of fibular fractures; and (iii) to introduce a new classification of tibial plateau fractures associated with proximal fibular fractures based on CT scan.

## Materials and Methods

Data were collected by reviewing all the patients who had been hospitalized for tibial plateau fractures in our trauma center between January 2010 and December 2014.

Inclusion criteria: (i) Patients suffering tibial plateau fractures associated with proximal fibular fractures; (ii) patients undergoing anterior–posterior and lateral X‐rays films, as well as CT films; (iii) patients have well‐documented medical.

Exclusion criteria: (i) pathologic fractures or old fractures; and (ii) patients did not have X‐rays films or CT films; (iii) patients younger than 12 years of age.

After exclusion, a total of 150 cases associated with fibular fractures in 502 tibial plateau fracture cases were identified from our institution database.

Three resident surgeons were trained to view all the radiographs on a clinical pictures and communication system (PACS). They first evaluated the radiographs to determine the type of tibial fracture and fibular fracture separately and then achieved a consensus together. All the tibial plateau fractures were classified on the basis of the Schatzker classification system. The cases were divided into two groups according to the number of condyles involved. Then the cases were further divided into five subgroups on the basis of fibular fracture type.

## Result

### *Classification of Proximal Fibular Fracture*

**Figure 1:**
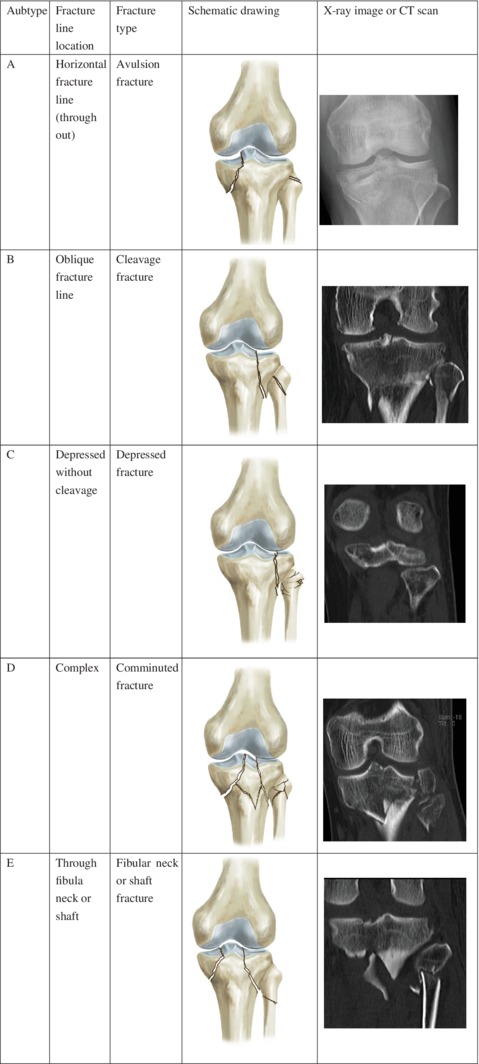
The new classification system of fibular fractures.

Our study group originally proposed a new classification system for fibular fractures, especially for cases that were additional to tibial plateau fractures. In this new classification system, fibular fractures are further divided into five subgroups according to fracture line and degree of comminution: (A) avulsion fibular head fracture with horizontal fracture line; (B) fibular head cleavage fracture with oblique fracture line penetrating into the fibular head; (C) fibular head depressed fracture, obviously depressed without cleavage on CT; (D) fibular head comminuted fracture with more than two fragments on CT; and (E) fibular neck or shaft fracture (Fig. 1).

### *Demographic Data*

A total of 150 cases were included in the study. There were 92 males and 58 females; the sex ratio was 1.60:1. The average age was 51 years (range, 14–78 years), including 1 case in the 11–20 years group, 18 cases in the 21–30 years group, 23 cases in the 31–40 years group, 29 cases in 41–50 years group, 45 cases in the 51–60 years group, 25 cases in the 61–70 years group, and 9 cases in the 71–80 years group. A total of 68 cases were left knee injuries and 82 cases are right‐side injuries. The mechanism of injury was a traffic accident in 69 cases, a fall from a height in 45 cases, simply a fall in 21 cases, an athletic injury in 7 cases, a crashing injury in 6 cases, a crush injury in 1 case, and a kick by a domestic animal in 1 case.

### *Fracture Patterns and Classification*

The incidence of fibular fractures in tibial plateau fractures was 29.88% (150/502); 71 cases (47.3%) involved one condyle and 79 cases (52.7%) involved both. The most common pattern in these case series was a split fracture with an oblique fracture line (type B, 32.67%, 49/150). The second most common pattern was a comminuted fracture (type D, 31.33%, 47/150). In the unicondylar group, the most common pattern was a split fracture with an oblique fracture line (type B, 38.03%, 27/71). In the bicondylar group, the most common pattern was a comminuted fracture (type D, 46.84%, 37/79). There was significant difference in the subgroup of avulsion fractures with a horizontal fracture line (Type A), with a ratio of 16.9% in the unicondylar group and 1.27% in the bicondylar group.

Details of the results of the fracture patterns and classification are summarized in Table 1.

**Table 1:** The results of the fracture patterns and classification [cases (%)]

Classifications	Unicondyle	Bicondyle	Total
A	12	1	13
	(16.90)	(1.27)	(8.67)
B	27	22	49
	(38.03)	(27.85)	(32.67)
C	16	11	27
	(22.54)	(13.92)	(18.00)
D	10	37	47
	(14.08)	(46.84)	(31.33)
E	6	8	14
	(8.45)	(10.13)	(9.33)
Total	71	79	150
	(100.00)	(100.00)	(100.00)

## Discussion

Tibial plateau fractures can range from a simple split fracture to a comminuted fracture, such as bicondylar injuries, which can result in severe soft tissue injuries^13^. Several authors report that severe fractures are associated with injuries such as collateral ligament injuries, compartment syndromes, meniscus injuries, and popliteal vascular injuries^14^, ^15^, ^16^, ^17^, ^18^. However, fibular fractures as an associated injury of tibial plateau fractures have rarely reported. In our study, the incidence of fibular fractures in tibial plateau fractures is 29.88% (150/502), which is similar to the incidence of cruciate ligament and collateral ligament injuries.

We suppose that the fracture patterns are the result of the injury mechanism. As the axial impact load is being transmitted from the femoral to the tibial articular surface, the lateral plateau depression fracture (S3) might be caused by the force during valgus moment. The fibular head depression (Type C) might be caused at the same time the tibial plateau depressed on the posterolateral area. The magnitude of the generated force increasing or the shearing force compressing the fibular head might be split (Type B) associated with lateral plateau fracture (S1, S2). While mixed force on the tibial plateau is always the reason for mixed fractures (S5, S6), fibular head comminuted fractures (Type D, Type E) always occur with bicondylar fractures and diastasis. The avulsion fibular head fracture (Type A) is a special case. This pattern always occurs as a result of an injury producing excessive varus forces coupled with axial loading (S4). In our study, 12 cases of avulsion fibular head fracture (Type A, 92.31% 12/13) were associated with medial tibial plateau fractures (S4). Huang *et al.* reported that the incidence of avulsion fractures of the fibular head was 0.6%, and the typical location of the avulsed osseous fragment was adjacent to the posterolateral rim of the tibial plateau^9^. Capps maintained that the avulsion fracture of the fibular head was commonly associated with proximal tibia fractures, as was the injury to the biceps femoris tendon and lateral collateral ligament^10^. However, Huang *et al*. indicated that there was no evidence of an avulsed bone fragment originating from the site of attachment of the lateral collateral ligament or the tendon of the biceps femoris muscle on MRI.

The integrity of the fibular head is important for the stability of the posterolateral corner (PLC) of the knee. In addition, the separation of the proximal tibiofibular joint in comminuted fibular head fractures (Type D in our study) and in bicondylar tibial plateau fractures may cause instability of the PLC. According to Capps *et al*.^8^, fibular head fractures are an easily missed injury of the knee. The vascular repair was emphasized by Green *et al*.^11^, who report on the high incidence (32%) of injuries to the popliteal artery that accompanies PLC injuries. However, in our study, no vascular injuries were observed. Ross *et al*. suggest that the lesions should be repaired within 2 weeks, to reduce the stability of the posterolateral corner^19^. Diagnosis of the separation of the proximal tibiofibular joint is difficult because it requires an awareness of the injury and eliciting the mechanism of injury. Certainly, injuries of the PLC were not evaluated by arthroscope in our study, and, thus, their overall impact on the clinical outcomes, if any, is unknown.

Whether surgical intervention for fibular fractures should be undertaken is highly controversial. Zhang presented a case of a 45‐year‐old man with a PCL injury and an arcuate avulsion fracture of the fibular head treated with a reduction of avulsed bone fragment and fixation with a suture anchor using an all‐arthroscopic technique^20^. Chung reviewed 6 cases of avulsion fracture of the fibular head associated with lateral instability of the knee with an average 2‐year follow‐up, supporting the effectiveness of surgical reduction and fixation^21^. In our clinical setting, we suggested reducing the fibular head before the reduction of the tibial plateau fracture preventing the instability of the PLC. The surgical technique was simple, involving anterolateraly inserting one or two K‐wires into the main fragment as a joystick and lifting the fibular head. The reduction is initially assessed by comparison of the radiographic views with C‐arm. In our limited experience, the reduction of fibular fractures, especially split fractures (type B), depressed fractures (Type C), and fibular shaft fractures (Type E), is helpful for the minimally invasive fixation of tibial plateau fractures and releasing excessive local strain of the lateral tibial plateau.

This study had several limitations. We would like to conclude that fibular head fracture is predominantly associated with tibial plateau fractures. However, long‐term follow up for functional outcome still needs to be obtained to further confirm the impact of fibular head fractures on PLC. Whether such a classification system will help to predict prognosis can only be determined by senior doctors in a further prospective clinical verification trial.

## *Conclusion*

This study mainly determined the incidence of fibular head fracture to be an associated injury of tibial plateau fractures. We proposed a new classification of this associated injury, describing the morphology of the fracture fragments. This classification system may improve the understanding of fibular head fractures as an associated injury of tibial plateau fractures, and help to enhance surgical plans and reduction strategies.
